# Effects of enzymatic reaction on the generation of key aroma volatiles in shiitake mushroom at different cultivation substrates

**DOI:** 10.1002/fsn3.2198

**Published:** 2021-02-26

**Authors:** Wen Li, Wan‐Chao Chen, Jin‐Bin Wang, Jie Feng, Di Wu, Zhong Zhang, Jing‐Song Zhang, Yan Yang

**Affiliations:** ^1^ Institute of Edible Fungi Shanghai Academy of Agricultural Sciences National Engineering Research Center of Edible Fungi Key Laboratory of Edible Fungi Resources and Utilization (South) Ministry of Agriculture Shanghai China; ^2^ Shanghai Guosen Bio‐tech Co. Ltd. Shanghai China; ^3^ Institute of Biotechnology Research Shanghai Academy of Agricultural Sciences Key Laboratory of Agricultural Genetics and Breeding Shanghai China

**Keywords:** aroma volatiles, cultivation substrate, enzymatic reaction, network model, shiitake mushroom, synthetase activity, synthetase gene expression

## Abstract

Aroma is an important factor affecting mushroom character and quality. According to the different reaction pathway, the key aroma metabolites (sulfur and eight‐carbon volatiles) formation can be classified into enzymatic reactions and nonenzymatic reactions. Aroma volatiles are generated from precursors via the biocatalytic activities of various synthases during the growth stages of shiitake mushrooms. Understanding the specific relationships between the key aroma metabolites and their synthases is key to improving shiitake mushroom quality. At the same time, to reduce forest logging and burning of agricultural by‐products in farmland, agricultural by‐products have been applied to shiitake mushroom cultivation. Nevertheless, how to further improve the production of aroma volatiles in mushroom cultivated with agricultural waste is still a challenge. In order to understand the biosynthesis of volatiles via enzymatic reactions and screen the agricultural by‐products that can improve the production of aroma volatiles in mushroom cultivation, the mechanism of producing aroma volatiles needs to be further elucidated. In this study, the activities and gene expression levels of the key synthases involved in volatile metabolism, the contents of key aroma volatiles, and the correlations between related synthetase, volatiles, and cultivation substrate (CS) were investigated. Network models for visualizing the links between synthetase, volatiles, and CSs were built through partial least squares (PLS) regression analysis. The correlation coefficients among three related synthetase and enzymatic gene expression were high, and the combined effects of multiple synthetase promoted the production of volatiles. PLS analysis showed that the corncob and corn meal were more related to the production of volatiles and synthetase gene expression, and they can be added to the CSs as flavor promoting substances. The enrichment of key aroma volatiles in shiitake mushroom cultivated by the gradient of 20% corn meal combination CS was noticeable.

## Graphical abstract

In this study, the biosynthesis of volatiles via enzymatic reactions, the agricultural by‐products that can improve the production of aroma volatiles in mushroom cultivation, and the mechanism of producing aroma volatiles were elucidated.

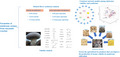

## 1 INTRODUCTION

The yield of mushrooms (edible fungi) is about 39.34 million tons (2019), and shiitake mushrooms (*Lentinula edodes*) hold the highest market share in China (http://www.cefa.org.cn). At present, the industrial cultivation of shiitake mushroom and the industrial preparation of mushroom cultivation sticks have also risen rapidly, and the quality and scale of the development of the shiitake mushroom industry has improved significantly (Huang & Zheng, 2020; Schimpf & Schulz, 2016). The large‐scale industrial production capacity of shiitake mushroom sticks is 20 million bags in 2018 (http://www.cefa.org.cn). With the continuous expansion of the production scale of shiitake mushroom, the demand for raw materials has increased significantly. Forestry is prohibited from cutting and logging, resulting in a shortage of the main raw materials (sawdust) for efficient use of mushroom cultivation. Therefore, increasing numbers of agricultural by‐products are being added to cultivation substrate (CS) formulations for mushroom cultivation (Atila, 2019; Lin et al., 2015). In China, the annual output of cottonseed hull can reach more than 2 million tons (http://www.china‐cotton.org/), bagasse is 12.58 million tons (Lin, 2020), and corncob is 45.9 million tons (Wang et al., 2016). Bagasse, corncob, and cottonseed hull have been gradually utilized as mushroom CSs. These agricultural by‐products have large yields and low costs, and the used CS can also be processed into biological fertilizers to form a virtuous ecological cycle.

The nutrients and unique aroma of shiitake mushroom are undoubtedly the main drivers for the increase in mushroom production/consumption. Aroma is an important factor affecting mushroom character and quality. Maintaining the flavor quality of shiitake mushrooms under the factory cultivation mode has become an important issue that needs to be studied for the shiitake mushroom industry in China. Several key aroma volatiles have been suggested to be important for the shiitake aroma. Sulfur and eight‐carbon volatiles are the main flavor compounds produced during the growth of shiitake mushroom (Beluha & Ranogajec, 2011; Chen et al., 2016; Dermiki et al., 2013; Her et al., 2015; Phat et al., 2016; Schmidberger & Schieberle, 2020; Tian et al., 2016; Yang et al., 2017), and play an important role in the flavor of shiitake mushroom. Sulfur compounds have a relative content of 19.41%–21.53% in fresh mushrooms and 37.51%–62.09% in dried mushrooms. The typical mushroom and grass aroma of fresh shiitake mushroom are mainly caused by the eight‐carbon compounds, and the most representative compounds are 1‐octen‐3‐ol, 1‐octen‐3‐one, and 3‐octanone. The relative content of eight‐carbon volatiles in fresh mushrooms can reach 44.13% and 4.4%–10.28% in dried mushrooms (Chen et al., 2018).

According to the different reaction pathway, the key aroma volatiles formation can be classified into enzymatic reactions and nonenzymatic reactions. In the enzymatic reaction, volatiles are generated from precursors via the biocatalytic activities of various synthetase during the growth stages of shiitake mushrooms. The synthetase plays a key role in the synthesis of volatiles on the enzymatic reaction biosynthetic pathway in mushrooms (Iwami et al., 1975; Li et al., 2018; Yasumoto et al., 1971). γ‐glutamyl transpeptidase (γ‐GGT), cysteine sulfoxide lyase (C‐S lyase), and lipoxygenase (LOX) were found to be involved in the synthesis and metabolism of sulfur and eight‐carbon volatiles. However, little is known about the relationship of the enzyme activity, aroma‐related genes expression with the production of key aroma volatiles in the enzymatic reaction during the growth and development of shiitake mushrooms. Therefore, the analysis of aroma‐forming of shiitake mushroom based on its aroma synthesis pathways could further clarify the corresponding mechanism of volatiles to the synthetase.

In this study, we have mostly focused on shiitake mushroom key aroma volatiles formation from enzymatic reactions. Firstly, we investigated into the contents of sulfur and eight‐carbon volatiles prevalent in mushrooms, which are derived from lentinic acid and unsaturated fatty acids (linoleic acid and linolenic acid). Secondly, the activity of related synthetase and its gene expression involved in the reactions were studied. Furthermore, network models were also built to represent the links between volatiles, synthetase, and CSs. The network models will clarify the corresponding mechanism of volatiles to synthetase, volatiles and synthetase to the CSs. The results can be used to guide and regulate the enzymatic reaction, resulting in abundant accumulation of the key aroma volatiles in shiitake mushroom during the growth and development stages.

## 2 MATERIALS AND METHODS

### 2.1 Mushroom cultivation

We developed more types of available carbon sources (such as corncob and cottonseed hull) which can replace the sawdust, and the addition ratio and gradient of the selected nitrogen sources (wheat bran and corn meal) for clarifying the aroma‐forming mechanism based on our previous study (Li et al., 2018, 2019). The shiitake mushroom CSs composition is listed in Table 1. Homogenized substrates were collected in mushroom bags with a moisture content of 55%, and the sterilized substrates were inoculated with colonized spawn from the selected strain L215 (strain certificate, 2,014,001). The mushrooms were cultivated in a factory cultivation mode (Figure S1). Fresh mushrooms were harvested at the mature growth stage, and the selected fresh mushroom pileus and stipes were separated for experimental use. The mushroom harvested at CS1–CS11 were expressed as P1–P11 for pileus samples and S1–S11 for stipe samples.

**TABLE 1:** Cultivation substrate material

Material	CS1	CS2	CS3	CS4	CS5	CS6	CS7	CS8	CS9	CS10	CS11
Sawdust^a^	39^c^	39	39	78	83	88	93	78	83	88	93
Bagasse^a^	39	0	0	0	0	0	0	0	0	0	0
Corncob^a^	0	39	0	0	0	0	0	0	0	0	0
Cottonseed hull^a^	0	0	39	0	0	0	0	0	0	0	0
Wheat bran^b^	20	20	20	20	15	10	5	0	0	0	0
Corn meal^b^	0	0	0	0	0	0	0	20	15	10	5
Gypsum	1	1	1	1	1	1	1	1	1	1	1
Sugar	1	1	1	1	1	1	1	1	1	1	1

^a^ Carbon sources.

^b^ Nitrogen sources.

^c^ Values are expressed as g per 100 g dry weight.

### 2.2 Extraction and analysis of volatiles

Mushroom volatiles were extracted by solid‐phase microextraction (SPME) and analyzed by gas chromatography–mass spectrometry (GC‐MS). The homogenized sample (10.0 g) was placed into the SPME headspace vial (20 ml), mixed with 5 ml deionized water and 10 µl 1,2‐dichlorobenzene (as an internal standard used for the semiquantification of volatiles, 100 mg/L, Sinopharm Chemical Reagent Co. Ltd.), and sealed. The SPME extraction and GC‐MS analysis conditions were carried out as described by Li et al., (2019). Each volatile compound was analyzed using authentic standards C7–C30 n‐alkanes (Anpel Laboratory Technologies Inc.), and linear retention time indices (RI) were determined. All of the mass spectra were analyzed and quantified by Agilent GC‐MS MSD ChemStation data analysis software (G1701EA E.01.00, Indah Medical Ltd) with NIST 05 and Wiley 6 mass spectrometry databases (Hewlett‐Packard Co.). The quantities of volatiles were calculated by the internal standard method (Chen et al., 2016), meanwhile the results were compared and corrected with the data calculated by volatile compound standard curves. The analytical standards employed for the positive identification of the volatiles with at least 98% purity were obtained from Sigma‐Aldrich. For unavailable standards, a tentative identification of the volatiles was achieved by comparing their mass spectrometric information of chromatographic peak with mass spectra library (similarity is greater than 75%), and manual analysis was used to identify the structure of the compound for qualitative analysis.

### 2.3 Activities of synthetase analysis

The activities of LOX, γ‐GGT, and C‐S lyase were studied to reveal the relationship between their activities and volatiles. The activities of LOX and γ‐GGT were analyzed as described by Li et al., (2019). LOX catalyzes the reaction of linoleic acid into oxidative products, which have a characteristic absorption peak at a wavelength of 280 nm. γ‐GGT catalyzes the transfer of γ‐glutamyl from glutamyl‐nitroanilide to N‐glycylglycine to form p‐nitroaniline, which has a characteristic absorption peak at a wavelength of 405 nm. C‐S lyase catalyzes the reaction of S‐methyl‐L‐cysteine sulfoxide to produce pyruvate, which reacts with 2,4‐dinitrophenylhydrazine. It appears brownish red under alkaline conditions and has a characteristic absorption peak at 510 nm. The values of absorbance were measured to calculate enzymatic activity. The three kinds of synthetase activity assay kits (Suzhou Comin Biotechnology Co., Ltd.) were used for LOX, γ‐GGT, and C‐S lyase enzymatic extraction and assays according to the manufacturer's instructions.

### 2.4 Gene expression of synthetase analysis

The mushroom RNA extraction and verification methods used were consistent with the literatures (Li et al., 2019; Liu et al., 2015), and the strand cDNA was obtained using a reverse transcription reaction system, following the manufacturer's instructions (Reverse Transcription System Technical Bulletin Kit, Promega Corporation).

The total RNA was extracted from mushroom samples using the Eastep Super Total RNA Extraction Kit (Promega Biotech Co., Ltd.). RNA concentration and quantity were determined using a NanoDrop Lite Spectrophotometer (ThermoFisher Scientific), and the RNA samples with the A260:A280 ratios between 1.8 and 2.2 were used for further analysis. First‐strand cDNA was synthesized from 1.0 μg of total RNA using a reverse transcription kit (Reverse Transcription System Technical Bulletin Kit, Promega Corporation). The synthesized cDNA was diluted 10 times with nuclease‐free water and stored at −20°C.

The relative expression level of LOX (GenBank accession no. GAW09948.1), GGT (GenBank accession no. JX123478.1), and C‐S lyase (GenBank accession no. AF126049.1) were detected with specific primer pairs. Primer pairs used for LOX, GGT and C‐S lyase gene evaluation were the following: LOX‐forward (5′‐GTGACCATCTTCAACCGTCG‐3′), LOX‐reverse (5′‐TGACGAGATGGATAGCGACC‐3′), GGT‐forward (5′‐GGAATCAACGATTGCGGACGAGAC‐3′), GGT‐reverse (5′‐CACCTGCACACAGGAAGACCCA‐3′), C‐S lyase‐forward (5′‐CTAGCTAGCATGTCCAATACACAATCCATCGCGC‐3′), and C‐S lyase‐reverse (5′‐CCGCTCGAGTCAAGGCCTAATGGAAGCAAGCTGC‐3′). The 18S gene (Genbank accession no. JN234840.1) served as an internal control. The conditions of real‐time quantitative PCR were 45 cycles of 95°C for 10 s and 62°C for 60 s. The fold change (relative quantity, RQ) of the target gene relative to the internal control was calculated using the formula RQ = 2^−ΔΔCt^.

### 2.5 Data analysis

Quantitative analysis of volatiles was performed in triplicate, and average values of concentration were used. Enzymatic gene expression results were processed by a one‐way analysis of variance (ANOVA) using SPSS Statistics 20.0 software (IBM Watson Analytics). The z‐score algorithm was used to standardize the content of the identified volatiles in the heat map, and the Euclidean distance average connection method was used to perform sample cluster analysis on the standardized data. The software used by the clustering algorithm was Python 2.7. Partial least squares (PLS) regression analysis was used to investigate the correlations between volatiles, synthetase, and CSs. PLSR was performed with IBM SPSS Statistics 20.0 software. Cytoscape 3.8.0 was used to construct the enzymatic regulation network, which reflected the relationships between synthetase and volatile compounds through line thicknesses and labels (positive, +; negative, ‐).

## 3 RESULTS

### 3.1 Analysis of volatiles

Five sulfur compounds and 12 types of eight‐carbon compounds were selected to study the relationship between the production of key aroma volatiles and CSs. The structural formula information, odorant description, and recognition threshold of volatiles are listed in Table 2, and their respective odor activity values (OAV) were calculated to estimate their contribution to the overall aroma. The selected volatiles, dimethyl trisulfide, 1‐octen‐3‐one, 1‐octen‐3‐ol, 1,2,4‐trithiolane, octanal, and dimethyl disulfide played an important role on the flavor profile of shiitake mushroom (OAV > 1).

**TABLE 2:** The information of structural formula, odorant description, and recognition threshold of volatile compounds

Volatile compounds	CAS No.	RI	Molecular formula	Structural formula	Odorant description^a^	Recognition threshold (mg/kg)^b^	OAV value in the mushroom pileus	OAV value in the mushroom stipe
Dimethyl disulfide	624–92–0	1,068	C_2_ H_6_ S_2_		Cabbage, onion	0.00006 in water	0 ~ 112.33^c^	0
Dimethyl trisulfide	3658–80–8	1,393	C_2_ H_4_ S_3_		Cooked onion, savory, meaty	0.000008 in water	263.75 ~ 3,967.50	0
1,2,4‐Trithiolane	289–16–7	1733	C_2_ H_4_ S_3_		Garlic flavor	0.00747 in water	3.36 ~ 24.58	2.85 ~ 42.57
1,2,4,5‐Tetrathiane	291–22–5	2,200	C_2_ H_4_ S_4_		Saute, scallion garlic	NF	NA	NA
1,2,4,5,7‐Pentathiocane	81531–39–7	2,454	C_3_ H_6_ S_5_		Dried mushroom	NF	NA	NA
1‐Octanol	111–87–5	1574	C_8_ H_18_ O		Waxy, green citrus, sweet floral, fatty, coconut	0.054 in water	0.04 ~ 0.09	0.03 ~ 0.11
1‐Octen−3‐ol	3391–86–4	1,477	C_8_ H_16_ O		Mushroom, earthy, oily, raw chicken	0.002 in water	3.67 ~ 40.85	7.26 ~ 37.06
2‐Octen−1‐ol	26001–58–1	866	C_8_ H_16_ O		Sweet floral	NF	NA	NA
1‐Octen−3‐one	4312–99–6	1,333	C_8_ H_14_ O		Herbal, mushroom, earthy, musty	0.000003 in water	333.33 ~ 1,490	183.33 ~ 950
2‐Octanone	111–13–7	1,319	C_8_ H_16_ O		Earthy, weedy, natural woody herbal	0.05 in water	0.02 ~ 0.17	0.02 ~ 0.24
3‐Octanone	106–68–3	1,285	C_8_ H_16_ O		Fresh herbal, lavender, sweet mushroom	1 in water	0.002 ~ 0.027	0.0008 ~ 0.039
3‐Octen−2‐one	1669–44–9	1,432	C_8_ H_14_ O		Earthy, spicy herbal, sweet Mushroom, hay, blueberry	0.14 in refined vegetable oil	0.01 ~ 0.06	0.004 ~ 0.03
2,7‐Octanedione	1626–09–1	1,370	C_8_ H_14_ O_2_		Not found	NF	NA	NA
Octanal	124–13–0	1,318	C_8_ H_16_ O		Waxy, citrus orange peel, green fatty	0.0001 in water	19.30 ~ 61.30	13.60 ~ 59.60
(*E*)−2‐Octenal	2548–87–0	1,448	C_8_ H_14_ O		Fresh cucumber, fatty, green herbal banana, waxy, green leaf	0.061 in sunflower seed oil	0.17 ~ 0.34	0.07 ~ 0.87
Octane	111–65–9	800	C_8_ H_18_		Gasoline	0.94 in refined olive oil	0 ~ 0.02	0
Octanoic acid	124–07–2	2061	C_8_ H_16_ O_2_		Fatty, waxy, cheesy	101 (pH 5.6 in water)	9.90E−06 ~ 5.6E05	4.26E−06 ~ 3.92E−0

Abbreviations: NF, not found; NA, not analyzed.

^a^ From the Good Scents Company Information System.

^b^ From the Compilations of Flavor Threshold Values in Water and Other Media (Second Enlarged and Revised Edition).

^c^ The OAV is defined as the ratio of the content of each volatile compound in the sample to its respective odor threshold.

Some new innovation CSs that were beneficial for the production of aroma volatiles in shiitake mushroom were found. With the change of the carbon source (sawdust, 78%–93%) and nitrogen source (corn meal, 20%–5%) gradients in the CS, the content of 1,2,4‐trithiane was highest at the ratio of 20% nitrogen source (183.63 µg/kg). It also reached relatively higher contents of 125.13 µg/kg under a 5% corn meal gradient, 154.11 µg/kg under a 5% wheat bran gradient. 1,2,4,5‐tetrathiane had higher and similar contents in the pileus harvested at the corn meal gradient of 20% and 10%, which were 41.74 µg/kg and 40.07 µg/kg, respectively. The content of dimethyl trisulfide reached the highest content (78.74 µg/kg) under the gradient of 20% corn meal. By comparing the change trend of sulfur compounds in the pileus harvested in CSs with mixed carbon source and the pileus cultivated with single carbon and nitrogen source at a different gradient, it can be found that the change of the carbon and nitrogen source gradient had a greater impact than the change of the carbon source type, and single carbon and nitrogen source had a better effect on the production of volatiles in mushroom pileus than a mixed carbon source. The significant change of sulfur volatiles in shiitake mushroom pileus was those harvested in CSs containing a single carbon and nitrogen source.

The total contents of eight‐carbon volatiles in the pileus harvested at CSs with corncob and cottonseed hull were similar (103.46 µg/kg and 102.14 µg/kg, respectively). The two substrates easily promoted the production of eight‐carbon compounds, and the contents were much higher than that in the pileus cultivated by adding the same proportion of bagasse in the CS (77.65 µg/kg). The content of 1‐octen‐3‐ol (7.33–81.69 µg/kg) and (*E*)‐2‐octenal (10.23–20.49 µg/kg) in mushroom pileus was higher. The content of 1‐octen‐3‐ol (81.69 µg/kg) and 2‐octen‐1‐ol (5.30 µg/kg) both reached the highest under a gradient of 20% corn meal, and 1‐octanol content reached a higher level under a gradient of 5% corn meal. (*E*)‐2‐octenal content in the pileus also reached the highest (20.49 µg/kg) when harvested from the corn meal gradient of 5%. The content of octane reached the highest (16.53 µg/kg) when the proportion of wheat bran in CS was 10%. The content of 3‐octanone in the pileus was highest (27.08 µg/kg) when the corn meal gradient was 20% in the CS. The content of other compounds was the highest under the gradient of 5% corn meal (2,7‐octanedione, 7.91 µg/kg; 3‐octen‐2‐one, 8.09 µg/kg; 1‐octene‐3‐ketone, 4.47 µg/kg).

In the stipe, the volatiles identified were reduced compared with those in the pileus, and dimethyl disulfide, dimethyl trisulfide, 1,2,4,5,7‐pentathiocane, 2,7‐octanedione, and octane were not detected. The identified volatiles of the stipe S1–S3 were all enriched in S1 except for (*E*)‐2‐octenal and 3‐octen‐2‐one, while (*E*)‐2‐octenal and 3‐octen‐2‐one were highly enriched in S3. Some volatiles had significantly high levels, such as 1,2,4‐trithiane, 1,2,4,5‐tetrathiane, 1‐octen‐3‐ol, and (*E*)‐2‐octenal. They were mainly distributed in the stipe cultivated in the wheat bran gradient 5% and the corn meal gradients 20% and 15% (S7, S8), of which 1,2,4‐trithiane (S7, 317.99 µg/kg), 1‐octene‐3‐ol (S8, 74.12 µg/kg), (*E*)‐2‐octenal (S8, 52.85 µg/kg), and 1,2,4,5‐tetrathiane (S8, 35.52 µg/kg) contents all exceeded the contents of the same compounds in the pileus P7, and the enrichment effect was noticeable. The main volatiles identified with contents higher than 35 µg/kg, including 1,2,4‐trithiane, 1,2,4,5‐tetrathiane, and 1‐octen‐3‐ol, were clearly enriched in the stipe S8 sample, and the content of these compounds was also higher in pileus P8.

**FIGURE 1:**
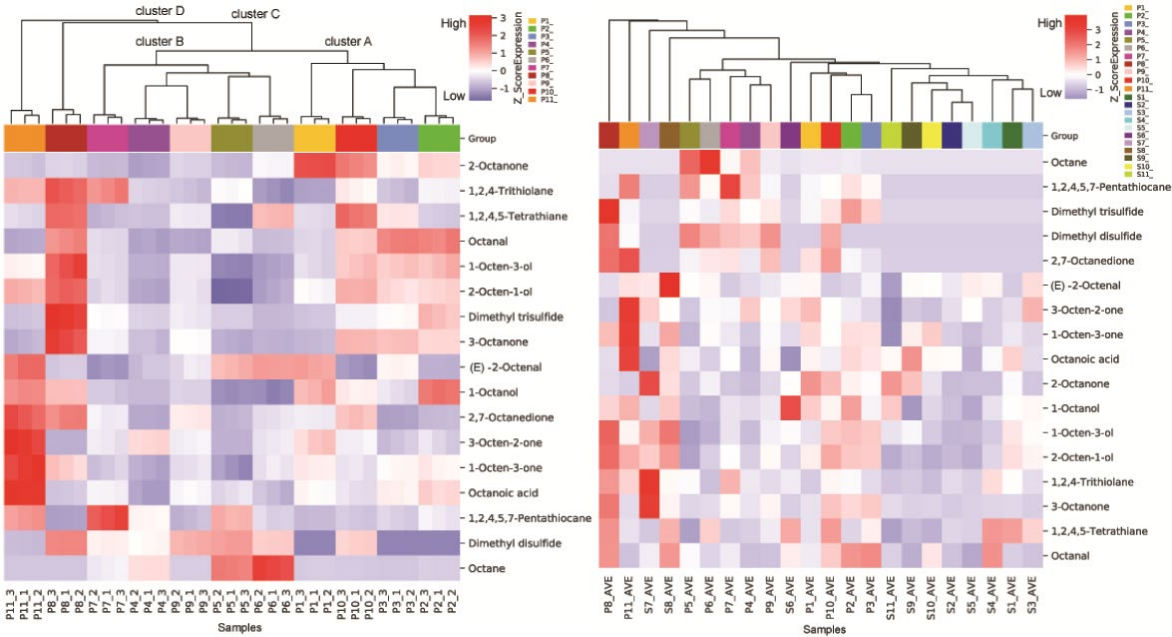
Heat map and hierarchical cluster analysis clustering results of selected volatile compounds in shiitake mushroom grown in different cultivation substrate formulations

To visualize the relationship between the shiitake mushroom grown in different CS formulations, a heat map and hierarchical cluster analysis (HCA) were used for the correlation, as shown in Figure 1. It can be seen from the cluster heat map that the pileus samples P2, P3, and P10 form the cluster A group, mainly because the CSs that promoted the volatiles octanal, 1‐octen‐3‐ol, 2‐octen‐1‐ol, and 3‐octanone synthesis were equivalent in these three pileus samples. Corncobs and cottonseed hulls had little difference in promoting the synthesis of the four volatiles in the pileus P2 and P3 samples, and the effects of the two substrates to promote the volatiles were better than adding the same proportion of bagasse in the substrate. Mushroom pileus P9 cultivated with 15% corn meal formed cluster B with pileus P4–P7. The enrichment of volatiles in pileus P8 and P11 was noticeable, and it was significantly different from the content of volatiles in other pileus samples, and these two pileus samples finally formed new clusters C and D with clusters A and B. Among the CSs with sawdust and corn meal by gradients changing, 20% and 5% of the corn meal of the CS had a better effect on promoting the formation of volatiles. From the heat map of volatiles in the stipe samples, it could be seen that some of the volatiles in the S7 and S8 samples were relatively high. Some eight‐carbon compounds were significant in the stipe S8 cultivated with 20% corn meal in the CS. The clustering results of stipe samples were irregular.

In summary, the use of a composite carbon source in the CS and the addition of corncobs or cottonseed hulls can promote the production of sulfur and eight‐carbon volatiles in the mushroom pileus samples, which is better than adding the same proportion of bagasse to the substrate. Gradient changes of sawdust and corn meal had a greater influence on the synthesis of volatiles, and the enrichment of volatiles in the mushroom cultivated by the 78% sawdust and 20% corn meal combination CS was noticeable.

### 3.2 Analysis of synthetase activities and gene expression

**FIGURE 2:**
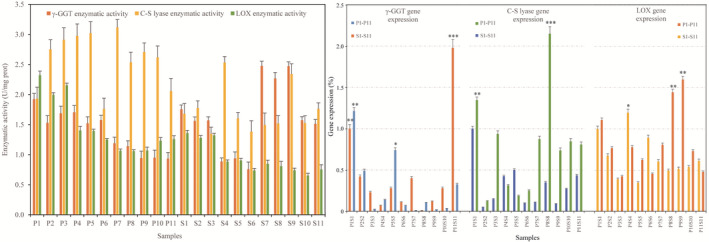
Enzymatic activity and mRNA levels of shiitake mushroom fruiting bodies grown in different cultivation substrate formulations

The shiitake mushroom fruiting bodies cultivated in different CSs had large differences in the activities of the three related synthetase and their distribution sites in the fruiting bodies. The enzymatic activities of γ‐GGT and LOX were higher in shiitake mushroom fruiting bodies cultivated by adding bagasse to the CS. In the mushroom pileus cultivated with the gradient of carbon and nitrogen sources in the CSs, the activities of the two enzymes showed a downward trend as the proportion of wheat bran decreased. With the decrease of the proportion of corn meal, the activity of γ‐GGT showed a slow downward trend, and LOX showed a slow upward trend. In the stipe, the activity of γ‐GGT was greatly improved at the ratio of 5% wheat bran gradient. As the gradient ratio of corn meal decreased, γ‐GGT was at a higher enzymatic activity level at the corn meal gradient of 20% and 15%, and then at the corn meal gradient of 10% and 5%, the activity of γ‐GGT decreased significantly. The activity of LOX in the stipe fluctuated with the change of the carbon and nitrogen source gradient of the CSs, and the enzyme activity did not change significantly. The enzymatic activities of C‐S lyase in the pileus were higher than those in the stipe harvested at the same CSs. The C‐S lyase had a relatively higher activities in the pileus cultivated with adding cottonseed hull and corncob to the composite carbon source. With the decrease of the proportion of wheat bran in CSs, it had higher enzymatic activities in the pileus harvested at the nitrogen source gradient of 20% and 15%, then it decreased significantly at the wheat bran gradient of 10%; then, its highest enzymatic activity was at the wheat bran gradient of 5%. In the stipe, the C‐S lyase enzymatic activity first decreased significantly at the nitrogen source gradients of 15% and 10%; then, it slightly increased at the ratio of 5% wheat bran gradient. With the decrease in the proportion of corn meal in CSs, C‐S lyase in mushroom pileus had a slight upward trend first at the corn meal gradient of 15% and then a downward trend at lower nitrogen source proportion, and the activity level of C‐S lyase in the stipe was also higher with corn meal at 15% in the CS (Figure 2).

The expression of three enzymatic genes was not completely consistent with their enzymatic activity. In the samples of mushroom pileus and stipe cultivated with the composite carbon source of bagasse added to the CS, the expression level of the γ‐GGT gene was higher. In the pileus, the change trend of the γ‐GGT gene expression with the reduction of wheat bran gradient first increased greatly when the wheat bran nitrogen source gradient was 15%, and as the wheat bran gradient further decreased, γ‐GGT gene expression dropped significantly. In the stipe, the expression level of the γ‐GGT gene was higher when the gradient of wheat bran was 15% and 5%. The expression of γ‐GGT gene in the pileus showed a trend of decreasing first and then increasing as the gradient of corn meal decreased, while the expression level of the γ‐GGT gene in the stipe showed an increasing trend as the gradient of corn meal decreased. The expression levels of γ‐GGT gene in the pileus and stipe samples were greatly improved when the corn meal gradient was 5%, and the expression levels of γ‐GGT gene in the stipes reached the highest value among all the collected samples when the corn meal gradient was 5%. The C‐S lyase gene had high expression in the pileus cultivated with adding bagasse to a composite carbon source, and the expression level of C‐S lyase gene was relatively higher compared with other two mushroom pileus samples cultivated with a composite carbon source. With the decrease in the proportion of wheat bran, the C‐S lyase gene expression in the pileus samples had a downward trend first and then an upward changing trend, and it was at a higher level with a nitrogen source ratio of 5%. In the stipe, the C‐S lyase gene expression first had an upward changing trend and then a downward trend, and it had higher levels at the wheat bran gradient of 15% and 20%. The C‐S lyase gene expression achieved its highest level in the mushroom pileus samples harvested at the CSs containing 20% corn meal, and then, it had a significantly decreased level as the corn meal gradient decreasing and presented a smooth changing trend at the low corn meal gradients. The changing trend of C‐S lyase gene expression showed a downward first and then an upward trend in the stipe as the corn meal gradient decreased in the CSs. The expression level of LOX gene was higher in the pileus and stipe cultivated with the composite carbon source of bagasse added to the CS, and the LOX expression was similar between the pileus and stipe samples harvested at the same CSs with a composite carbon source. With the decrease of wheat bran gradient in the CSs, the LOX expression in mushroom pileus showed first a downward and then an upward trend, and it had high levels in the pileus samples cultivated in CS with 20% and 5% wheat bran gradients. The LOX expression levels were higher in the stipe samples harvested in the CS with 20% and 10% wheat bran gradients, and they were higher compared with those in the pileus cultivated in the same CSs. The LOX expression levels were higher in the pileus samples harvested at CSs with 20% and 15% corn meal gradients, and then, it decreased as the corn meal proportion decreased. The LOX expression in the stipe changed slightly with the decreasing of corn meal gradients in the CSs (Figure 2).

**FIGURE 3:**
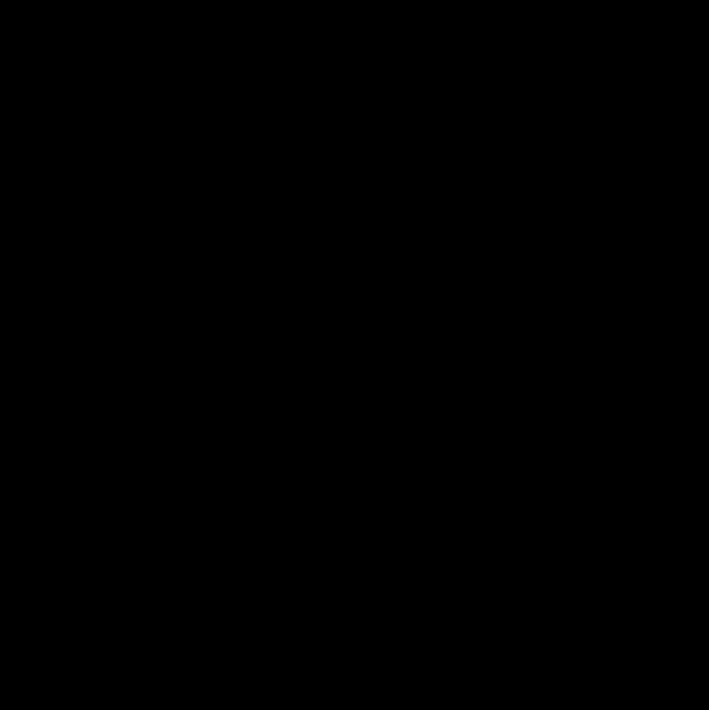
Network diagram of correlations between volatiles, synthetase, and CSs

According to Pearson correlation coefficients, there were high correlation coefficients among γ‐GGT activity and LOX activity (0.753), γ‐GGT expression and LOX activity (0.583), C‐S lyase expression and LOX expression (0.523), γ‐GGT activity and γ‐GGT expression (0.516). It can be seen that the synthetase and its gene expression had a synergistic effect on the production of aroma volatiles. Based on the correlation analysis of the synthetase and volatiles, most eight‐carbon ketones had a high correlation coefficient with γ‐GGT activity and γ‐GGT expression. There were high correlation coefficients between eight‐carbon aldehydes, alcohols, sulfur volatiles and the C‐S lyase gene expression level and enzymatic activity. LOX was mainly involved in the synthesis and metabolism of ketones and sulfur volatiles (Figure 3). It was speculated that the volatile formation is complex and diverse, and involving different enzyme‐catalyzed reactions between the substrate and enzymes.

### 3.3 Analysis of volatiles and synthetase responses mechanism to the CS

The PLSR method was employed to formulate mathematical models that allow the establishment of the relationships between the volatiles, synthetase, and CSs. In the proportion of variance explained for volatiles, synthetase and CS contribution, the x variables (*R^2^X* = 0.891) explained the variation in the y variables (*R^2^Y* = 0.952) according to the first three factors. PLS regression parameters are shown in Table S1 (Supplementary Material), and the correlation network model based on the PLS regression parameters was shown in Figure 3. The analysis results showed that the sawdust was highly correlated with some volatiles synthesis, such as 1,2,4‐trithiolane, octane, dimethyl disulfide, 1,2,4,5,7‐pentathiocane, and (*E*)‐2‐octenal. The change of carbon source type had a greater influence on the synthesis of 2‐octanone, (*E*)‐2‐octenal, 2,7‐octanedione, octanoic acid, 1‐octanol, 1‐octen‐3‐one, 3‐octanone, 2‐octen‐1‐ol, 1‐octen‐3‐ol, C‐S lyase activity, 1,2,4,5‐tetrathiane, LOX activity, γ‐GGT expression, and C‐S lyase expression in the CS with the addition of bagasse. With the addition of corncob in CS, the variety of carbon sources was highly correlated with the synthesis of 1,2,4‐trithiolane, 1‐octen‐3‐ol, dimethyl trisulfide, 1‐octanol, octanoic acid, octanal, 2‐octanone, 2‐octen‐1‐ol, 2,7‐octanedione, 1‐octen‐3‐one, 3‐octen‐2‐one, and 1,2,4,5,7‐pentathiocane. In composite carbon source CS added with cottonseed hull, the change of carbon source type had a greater effect on the synthesis of 1‐octen‐3‐ol, 1,2,4,5‐tetrathiane, octanal, octanoic acid, 2‐octen‐1‐ol, (*E*)‐2‐octenal, 2‐octanone, 1‐octen‐3‐one, 1‐octanol, 2,7‐octanedione, 1,2,4‐trithiolane, C‐S lyase expression, and LOX activity. In CSs with wheat bran, the change in the proportion of nitrogen source was highly correlated with the synthesis of octane, dimethyl trisulfide, dimethyl disulfide, γ‐GGT activity, and LOX expression; with the addition of corn meal in CSs, the ratio change of the corn meal had a greater effect on the synthesis of 1‐octen‐3‐ol, dimethyl trisulfide, 1,2,4‐trithiolane, 1,2,4,5‐tetrathiane, dimethyl disulfide, octanal, LOX expression, C‐S lyase expression, 2‐octen‐1‐ol, and 2,7‐octanedione. If the content of volatiles was not high or there was mutual conversion between compounds, the correlations between volatiles and matrix changes were low. It was also speculated that various types of synthetase, such as hydroperoxide lyase and alcohol oxidoreductase, participated in the synthesis of volatiles, or flavor synthetase also participated in the synthesis of other ingredients, which resulted in the complex correlation between volatiles, synthetase and substrate changes.

## 4 DISCUSSION

Japanese scholars discovered that lentinic acid formed the characteristic volatiles of lenthionine under the action of γ‐GGT and C‐S lyase, accompanied by by‐products (Iwami et al., 1975; Yasumoto et al., 1971). Combet et al., (2006) had also reviewed the formation pathway of eight‐carbon volatiles in fungi. Therefore, based on the synthetic pathway of characteristic volatiles of shiitake mushroom, our research results can provide a guide that can be used to regulate the synthesis of characteristic volatiles during the factory cultivation process by analyzing the response mechanism of volatiles synthesis and synthetase changes to the CS.

Masakazu et al., (2006), Masakazu et al., (2010) had studied the smell and odorous components of dried shiitake mushroom and found rice bran was the main source of sulfur, or added amino acids to sawdust media, which both had the most effect on 1,2,4‐trithiolane content. In our study, the correlation coefficient of 1,2,4‐trithiane and corn meal was the highest, followed by corncob. These results provided practical basis for the development of mushroom CSs.

Scholars in some Asian countries have studied the correlation between shiitake cultivation and mushroom flavor. Asia is the main area for shiitake mushroom cultivation and consumption, and research scholars from various countries had also done a lot of work on the yield production of mushrooms (Azman et al., 2019; Barshteyn & Krupodorova, 2016; Ozcelik & Pesken, 2007; Philippoussis, 2009; Rigoberto et al., 2020; Rossi et al., 2003), or investigated the flavor of shiitake mushrooms during processing (Tian et al., 2016; Wen et al., 2020; Xu et al., 2019). Along with flavor enhancement or agricultural recycling and resource development, there is still a lot of work to be done in the fields of mushroom cultivation and flavor quality research in the future.

## 5 CONCLUSIONS

The results showed that shiitake mushroom aromas, and their biosynthesis, are various and complex. The combined effects of multiple synthetase promoted the production of volatiles, and shitake mushroom had its own complex networks of volatile biosynthesis. γ‐GGT and its encoding genes were involved in the biosynthesis of eight‐carbon ketones, C‐S lyase and genes closely related to eight‐carbon aldehydes, alcohols, sulfur volatiles formation. Enzymatic assay also confirmed that LOX had positive effects on aroma volatiles production. The large variety of synthetase contributing to mushroom aroma in enzymatic reaction suggest that more remains to be learned. The carbon and nitrogen source material base and the addition ratio in the CS also had an important influence on the synthesis of characteristic volatiles in shiitake mushroom. On the CS containing corncob, it was easier to promote the production of the sulfur and eight‐carbon volatiles. Also, adding corn meal to the CS promoted better production of these volatiles and synthetase gene expression. The recommended order for the addition of carbon source to the CS is corncob, cottonseed hull, and bagasse; nitrogen source order is corn meal, wheat bran; the nitrogen source gradient order is 20% corn meal, 5% wheat bran, and 5% corn meal. In summary, as optimized and more environmentally friendly cultivation substrate constituents, corncob and corn meal can be added to the CSs as flavor promoting substances for the industrial cultivation of shiitake mushroom to obtain mushroom fruiting bodies rich in flavor compounds.

## CONFLICT OF INTEREST

The authors declare no conflict of interest.

## Supporting information

App S1
