# Supplementary material for: Effects of enzymatic reaction on the generation of key aroma volatiles in shiitake mushroom at different cultivation substrates
Source: Food Sci Nutr. 2021 Feb 26;9(4):2247–56. doi: 10.1002/fsn3.2198 (PMC8020957; doi:10.1002/fsn3.2198)
Supplement: Supplementary file 1 — App S1 [file FSN3-9-2247-s001.docx]

Appendix S1

Supplementary material-Tables and Figures

Table S1. The correlation parameters of the volatile compounds, synthetase and the cultivation substrate attributes.

| Dependent Variables | Constant | Independent Variables | | | | | |
| --- | --- | --- | --- | --- | --- | --- | --- |
|  |  | Sawdust | Bagasse | Corncob | Cottonseed hull | Wheat bran | Corn meal |
| Dimethyl disulfide | -0.711 | 0.032 | -0.103 | -0.103 | -0.103 | 0.175 | 0.233 |
| Dimethyl trisulfide | 10.352 | -0.118 | -0.186 | 0.107 | -0.041 | 0.238 | 1.051 |
| 1,2,4-Trithiolane | 88.420 | 0.201 | -0.375 | 0.743 | 0.018 | -2.405 | 0.978 |
| 1,2,4,5-Tetrathiane | 25.864 | -0.087 | 0.017 | -0.033 | 0.231 | -0.367 | 0.859 |
| 1,2,4,5,7-Pentathiocane | 1.092 | 0.021 | 0.000 | 0.015 | 0.007 | -0.070 | -0.147 |
| 1-Octanol | 4.909 | -0.014 | 0.044 | 0.061 | 0.019 | -0.108 | -0.017 |
| 1-Octen-3-ol | 60.216 | -0.372 | 0.026 | 0.671 | 0.600 | -1.006 | 1.740 |
| 2-Octen-1-ol | 4.725 | -0.018 | 0.026 | 0.043 | 0.040 | -0.108 | 0.060 |
| 1-Octen-3-one | 3.354 | -0.004 | 0.035 | 0.033 | 0.033 | -0.116 | -0.045 |
| 2-Octanone | 6.690 | -0.035 | 0.152 | 0.050 | 0.033 | -0.146 | -0.049 |
| 3-Octanone | 0.562 | 0.002 | 0.027 | 0.001 | 0.001 | -0.035 | -0.041 |
| 3-Octen-2-one | 0.732 | 0.003 | 0.001 | 0.027 | 0.001 | -0.044 | -0.031 |
| 2,7-Octanedione | 4.622 | 0.005 | 0.067 | 0.035 | 0.018 | -0.246 | 0.031 |
| Octanal | 5.224 | -0.031 | -0.024 | 0.052 | 0.056 | -0.010 | 0.091 |
| (*E*)-2-Octenal | 14.969 | 0.014 | 0.110 | -0.058 | 0.033 | -0.098 | -0.199 |
| Octane | -5.151 | 0.093 | -0.182 | -0.177 | -0.189 | 0.446 | -0.157 |
| Octanoic acid | 4.075 | 0.000 | 0.046 | 0.056 | 0.050 | -0.174 | -0.120 |
| γ-GGT activity | 1.252 | -0.003 | 0.001 | -0.009 | -0.005 | 0.037 | 0.000 |
| γ-GGT expression | 0.360 | -0.002 | 0.014 | 0.001 | -0.011 | 0.008 | -0.005 |
| C-S lyase activity | 0.331 | -0.003 | 0.022 | -0.003 | -0.003 | -0.003 | -0.003 |
| C-S lyase expression | 1.101 | -0.006 | 0.013 | -0.010 | 0.011 | -0.018 | 0.049 |
| LOX activity | 1.999 | -0.008 | 0.015 | 0.007 | 0.011 | 0.003 | -0.012 |
| LOX expression | 0.783 | -0.004 | 0.002 | -0.004 | -0.013 | 0.015 | 0.054 |


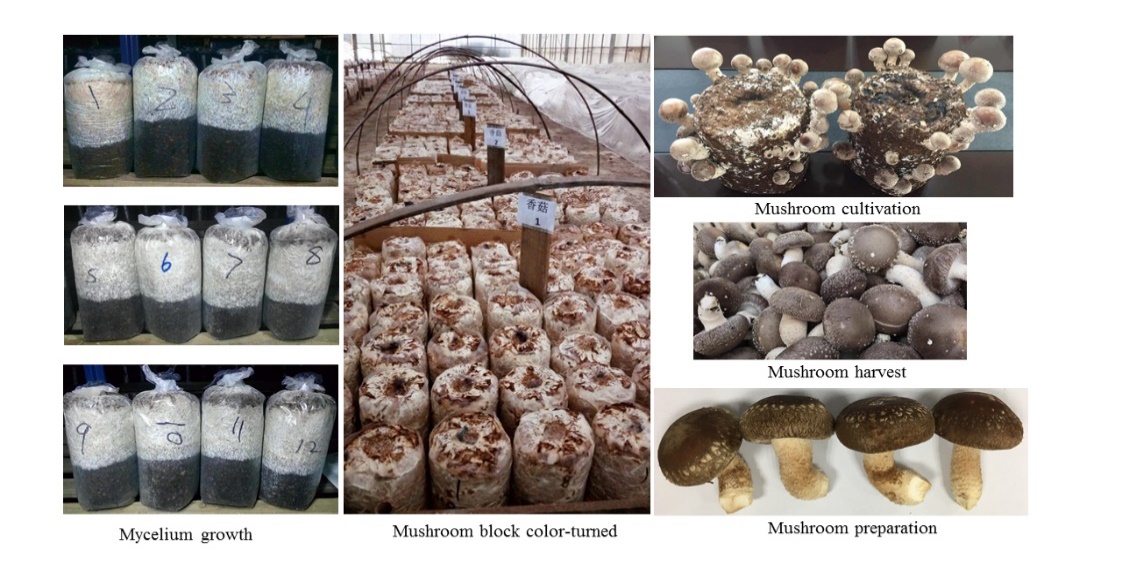


Figure S1. shiitake mushroom cultivation, harvest, and preparation.
